# Conformation-dependent ligand hot spots in the spliceosomal RNA helicase BRR2

**DOI:** 10.1107/S2059798323001778

**Published:** 2023-04-28

**Authors:** Karen Vester, Alexander Metz, Simon Huber, Bernhard Loll, Markus C. Wahl

**Affiliations:** a Laboratory of Structural Biochemistry, Institute of Chemistry and Biochemistry, Freie Universität Berlin, Takustrasse 6, 14195 Berlin, Germany; b Drug Design Group, Institute of Pharmaceutical Chemistry, Philipps-Universität Marburg, Marbacher Weg 6, 35032 Marburg, Germany; c Macromolecular Crystallography, Helmholtz-Zentrum Berlin für Materialien und Energie, Albert-Einstein Strasse 15, 12489 Berlin, Germany; Thomas Jefferson University, USA

**Keywords:** binding modes, conformation, drug discovery, fragment screening, ligand hot spots, RNA helicase BRR2

## Abstract

The conversion of hits to leads in drug discovery involves the elaboration of chemical core structures to increase their potency. In fragment-based drug discovery, low-molecular-weight compounds are tested for protein binding and are subsequently modified, with the tacit assumption that the binding mode of the original hit will be conserved among the derivatives. However, deviations from binding mode conservation are rather frequently observed, but potential causes of these alterations remain incompletely understood. Here, two crystal forms of the spliceosomal RNA helicase BRR2 were employed as a test case to explore the consequences of conformational changes in the target protein on the binding behaviour of fragment derivatives. The initial fragment, sulfaguanidine, bound at the interface between the two helicase cassettes of BRR2 in one crystal form. Second-generation compounds devised by structure-guided docking were probed for their binding to BRR2 in a second crystal form, in which the original fragment-binding site was altered due to a conformational change. While some of the second-generation compounds retained binding to parts of the original site, others changed to different binding pockets of the protein. A structural bioinformatics analysis revealed that the fragment-binding sites correspond to predicted binding hot spots, which strongly depend on the protein conformation. This case study offers an example of extensive binding-mode changes during hit derivatization, which are likely to occur as a consequence of multiple binding hot spots, some of which are sensitive to the flexibility of the protein.

## Abstract 2

Fragment derivatives occupy diverse binding sites in two crystal forms of the RNA helicase BRR2 that coincide with predicted, conformation-dependent ligand hot spots.

## 1. Introduction

Fragment-based drug discovery (FBDD) involves as an initial step the screening of small libraries of fragments of comparably low molecular weight (typically below 300 Da) for binding to a target protein (Scott *et al.*, 2012 ▸; Congreve *et al.*, 2003 ▸). FBDD offers an alternative to the high-throughput screening of large collections of higher molecular-weight compounds based on *in vitro* activity, binding or biological effects. Due to their low molecular weight and reduced combinatorial complexity with respect to chemical functional­ities, fragments show higher probabilities of protein binding and can be used to probe chemical space more efficient­ly and densely than is possible with larger compounds (Leach *et al.*, 2006 ▸). Furthermore, the optimization of fragments usually gives rise to leads with a lower lipophilicity, higher water solubility and improved cell permeability, whereas high attrition rates are often observed during the optimization of higher molecular-weight compounds (Murray *et al.*, 2012 ▸; Lipinski *et al.*, 2001 ▸; Teague *et al.*, 1999 ▸; Congreve *et al.*, 2003 ▸). As the affinity of fragments to target proteins is typically low (usually in the range 0.1–10 m*M*), sensitive biophysical methods are required for the detection of fragment binding (Schiebel *et al.*, 2016 ▸). In recent years, X-ray crystallography has emerged as one of the preferred methods for detecting initial fragment hits, as it (i) can nowadays be conducted in a high-throughput format, (ii) allows the identification of weakly binding substances due to the high substance concentrations that can be employed and (iii) provides immediate insights into the binding poses of the compounds with the target protein (Schiebel *et al.*, 2016 ▸; Chilingaryan *et al.*, 2012 ▸).

During elaboration of the initial hits, the binding mode of analogues with the same core scaffold is expected to be retained. A conserved binding mode is indeed observed in many cases (Murray *et al.*, 2012 ▸). For example, fragments derived from the chitinase inhibitor argifin (Andersen *et al.*, 2008 ▸) and fragments binding to the Src kinase showed the same interaction modes in structures compared with the intact inhibitors (Lange *et al.*, 2003 ▸). However, conservation of the binding modes is not always observed in inhibitor-to-fragment deconstruction studies. For example, after the deconstruction of nine inhibitors of Bcl-xL into 22 fragments, three of the fragments showed no interaction with the protein, the binding of six fragments could not be detected via NMR chemical shift changes and one fragment led to chemical shift changes in different regions of the protein, suggesting that it bound at a different position (Barelier *et al.*, 2010 ▸). A particularly remarkable example is afforded by the deconstruction of an inhibitor of the AmpC β-lactamase into three fragments, none of which bound to the original position in crystal structures (Babaoglu & Shoichet, 2006 ▸). More recently, 21 fragments were generated from three inhibitors of the HIV-1 reverse transcriptase. Only nine of them could be detected as binders in surface plasmon resonance (SPR) measurements; however, all of these compounds were above the typical fragment size, whereas all fragment-sized molecules did not interact (Brandt *et al.*, 2011 ▸). Likewise, the binding of fragments derived from an MDM2 inhibitor, nutlin, to the target protein was not detected when probed by SPR and NMR, and only larger portions resulted in detectable binding affinities (Fry *et al.*, 2013 ▸). Furthermore, the decomposition of the substrates of six enzymes into 41 compounds did not result in detectable binding or inhibitory activity for most of the fragments (Barelier *et al.*, 2014 ▸).

To identify features that contribute to changed binding modes, Malhotra and coworkers conducted a large-scale analysis of Protein Data Bank (PDB) entries for proteins in complex with ligands or in complex with smaller substructures, observing changed binding modes with a surprisingly high frequency (14%; Malhotra & Karanicolas, 2017 ▸). The analysis suggested that compounds with lower molecular weight, weaker protein binding, higher polarity and higher rigidity were more prone to show binding-mode alterations upon enlargement (Malhotra & Karanicolas, 2017 ▸). In another large-scale study of PDB structures, Drwal and coworkers noted an average conservation of above 80% of the binding modes of smaller ligands compared with the binding modes of larger ligands with similar (but not identical) substructures in almost 75% of the compared pairs (Drwal *et al.*, 2018 ▸). The authors further reported that the binding mode tends to be conserved for fragments with a molecular weight above 150 Da (Drwal *et al.*, 2018 ▸). Increased flexibility of the protein in the ligand-binding site was thought to contribute to changes in binding modes (Drwal *et al.*, 2018 ▸). In an attempt to analyse the protein properties that are associated with changed fragment-binding modes, Kozakov and coworkers surveyed the overlap of fragment-binding sites with binding hot spots, *i.e.* regions contributing a substantial amount of free energy upon binding, and suggested that the strength of a hot spot and its overlap with a fragment-binding site are critical for binding-mode conservation (Kozakov, Hall, Jehle *et al.*, 2015 ▸).

**Figure 1:**
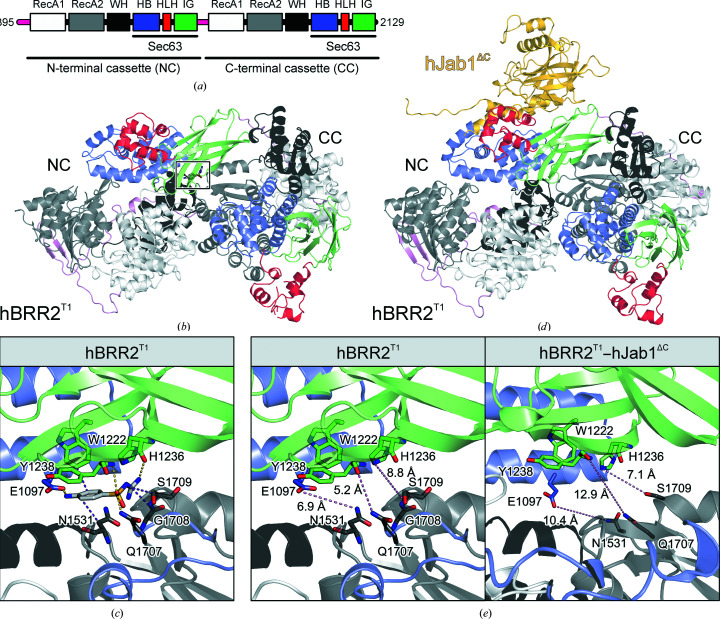
Crystal structures of hBRR2^T1^ and the binding site of sulfaguanidine. (*a*) Domain scheme of hBRR2^T1^. hBRR2^T1^ comprises tandem helicase cassettes (NC, N-terminal helicase cassette; CC, C-terminal helicase cassette), each containing dual RecA-like domains (RecA1 and RecA2), a winged-helix (WH) domain, a helical bundle (HB) domain, a helix–loop–helix (HLH) domain and an immunoglobulin-like (IG) domain. The HB, HLH and IG domains form Sec63 homolog units. (*b*) Crystal structure of isolated hBRR2^T1^ in complex with sulfaguanidine (boxed). Unless mentioned otherwise, in this and the following figures or figure panels, hBRR2 domains are coloured according to the scheme in (*a*). (*c*) Sulfaguanidine binding pocket in isolated hBRR2^T1^. Residues of hBRR2^T1^ involved in the binding of sulfaguanidine are shown as sticks and are coloured by atom type. Unless mentioned otherwise, in this and the following figures or figure panels, protein C atoms are coloured as the respective protein region, compound C atoms in light grey, N atoms in blue, O atoms in red and S atoms in yellow; violet dashed lines represent van der Waals interactions or π-stacking interactions and yellow dashed lines represent hydrogen bonds. (*d*) Crystal structure of the hBRR2^T1^–hJab1^ΔC^ complex (PDB entry 6s8q). Unless mentioned otherwise, in this and the following figures or figure panels, hJab1^ΔC^ is shown in gold. (*e*) Selected interatomic distances between residues of the NC and the CC in the hBRR2^T1^–sulfaguanidine complex structure (left panel) and in the hBRR2^T1^–hJab1^ΔC^ structure (right panel) are shown as pink dashed lines. Bound sulfaguanidine was omitted from the left panel.

Here, we report a test case in which we probed the binding of compounds derived from an initial fragment hit for the spliceosomal RNA helicase BRR2 in one crystal form to the protein with an altered conformation in another crystal form. BRR2 is responsible for the unwinding of U4/U6 di-small nuclear (sn) RNAs, an essential step for the conversion of a pre-catalytic spliceosome (B complex) to an activated spliceo­some (B^act^ complex; Raghunathan & Guthrie, 1998 ▸; Laggerbauer *et al.*, 1998 ▸). The ∼250 kDa enzyme is composed of an N-terminal auto-regulatory region of about 400 residues and tandem helicase cassettes, each comprising two RecA-like domains (RecA1 and RecA2), a winged-helix (WH) domain, a helical bundle (HB) or ratchet domain, a helix–loop–helix (HLH) domain and an immunoglobulin-like (IG) domain (Absmeier *et al.*, 2015 ▸; Santos *et al.*, 2012 ▸; Pena *et al.*, 2009 ▸; Fig. 1 ▸ *a*). Due to its essential role in each splicing event and its association with the genetically inherited visual deficiency retinitis pigmentosa (Zhao *et al.*, 2009 ▸; Ledoux & Guthrie, 2016 ▸; Růžičková & Staněk, 2017 ▸), BRR2 is an interesting target for the development of small-molecule modulators. In our study, nine fragment derivatives with the same core structure showed drastic alterations in their binding modes or locations, occupying six different pockets on the protein upon conformational changes affecting the original fragment-binding site. Some of the fragment derivatives could be interesting starting points for further optimization into novel splicing modulators. Our study provides a test case for fragment elaboration on a large protein with a high number of potential ligand-binding pockets and high intrinsic flexibility.

## 2. Methods

### 2.1. Protein production and purification

Proteins were produced via recombinant baculoviruses in insect cells as described previously (Mozaffari-Jovin *et al.*, 2013 ▸; Vester *et al.*, 2020 ▸). Briefly, bacmids encoding an N-terminally truncated human BRR2 (hBRR2) variant (residues 395–2129; hBRR2^T1^) fused to an N-terminal TEV-cleavable His tag or to a C-terminal Jab1/MPN region of the hPRPF8 protein lacking a hBRR2-inhibitory C-terminal tail (residues 2064–2320; hJab1^ΔC^) fused to an N-terminal PreScission-cleavable GST tag were transfected into Sf9 cells for the production of first virus generations. The viruses were used to infect Sf9 cells to generate the second virus generations. Proteins were produced on a large scale upon the infection of High Five cells with the second generation of viruses.

hBRR2^T1^ and hJab1^ΔC^ proteins were produced and purified as described previously (Mozaffari-Jovin *et al.*, 2013 ▸; Vester *et al.*, 2020 ▸). Briefly, hBRR2^T1^ was captured on a HisTrap column (GE Healthcare), eluted, dialyzed overnight including His-tag cleavage, passed over a HisTrap column again, treated with RNase and further purified via a HiPrep Heparin column (GE Healthcare). Finally, the protein was subjected to size-exclusion chromatography (SEC) on a HiLoad Superdex 200 16/60 column (GE Healthcare) in 10 m*M* Tris–HCl pH 7.5, 200 m*M* NaCl, concentrated to 10 mg ml^−1^, aliquoted and flash-frozen in liquid nitrogen. For the hBRR2^T1^ protein used for activity assays, the His tag was not cleaved and the final SEC step was conducted with 10 m*M* Tris–HCl pH 7.5, 200 m*M* NaCl, 10%(*v*/*v*) glycerol. The hJab1^ΔC^ protein was purified via glutathione (GSH) beads (GE Healthcare), buffer-exchanged by SEC, dialyzed overnight including cleavage of the GST tag and passed over GSH beads again. After SEC on a Superdex 75 16/60 column (GE Healthcare) in 10 m*M* Tris–HCl pH 8.0, 150 m*M* NaCl, the protein was concentrated to 4 mg ml^−1^, aliquoted and flash-frozen in liquid nitrogen. The hBRR2^T1^–hJab1^ΔC^ complex was formed by mixing purified hBRR2^T1^ with a 1.5-molar excess of purified hJab1^ΔC^, followed by SEC on a Superdex 200 10/300 Global Increase column (GE Healthcare) in 20 m*M* Tris–HCl pH 8.0, 150 m*M* NaCl, concentrating to 6 mg ml^−1^, aliquoting and flash-freezing in liquid nitrogen.

### 2.2. Crystallographic procedures

The crystallographic work was based on previously reported crystals of hBRR2^T1^ (Santos *et al.*, 2012 ▸) and of the hBRR2^T1^–hJab1^ΔC^ complex (Vester *et al.*, 2020 ▸). Briefly, hBRR2^T1^ was crystallized by sitting-drop vapour diffusion with drops consisting of 1 µl hBRR2^T1^ solution (10 mg ml^−1^) and 1 µl reservoir solution (0.1 *M* sodium citrate, 1.5 *M* sodium malonate pH 7.0). The hBRR2^T1^–hJab1^ΔC^ complex was crystallized by sitting-drop vapour diffusion with drops consisting of 1 µl hBRR2^T1^–hJab1^ΔC^ complex (6 mg ml^−1^) and 1 µl reservoir solution [0.1 *M* HEPES–NaOH pH 8.0, 0.1 *M* MgCl_2_, 8%(*w*/*v*) PEG 3350].

The initial sulfaguanidine [4-amino-*N*-(aminoiminomethyl)benzolsulfonamide] hit was obtained by co-crystallization of hBRR2^T1^ with a cocktail of additives (Silver Bullets condition 12; Hampton Research; added to the crystallization drop at 1/10 volume) and was subsequently validated by co-crystallization with sulfaguanidine alone (50 m*M* final concentration in the crystallization drop). Apart from the addition of the additive cocktail or of sulfaguanidine, co-crystallization was conducted as for isolated hBRR2^T1^ crystals. After cryoprotection with 0.1 *M* sodium citrate, 3.0 *M* sodium malonate pH 7.0, 0.1 *M* NaCl, crystals were flash-cooled in liquid nitrogen for subsequent diffraction data collection.

Both co-crystallization in the presence of fragment derivatives and the soaking of preformed hBRR2^T1^ crystals with fragment derivatives were tested to obtain additional fragment derivative-bound hBRR2^T1^ crystal structures. For co-crystallization of isolated hBRR2^T1^ with fragment derivatives, small-molecule stock solutions were combined with the protein solution in a 1:9 volume ratio so that the final DMSO concentration was 10%(*v*/*v*). For soaking experiments, preformed hBRR2^T1^ crystals were incubated with varying concentrations of fragment derivatives and for different times. Neither co-crystallization nor soaking led to additional fragment-bound hBRR2^T1^ structures (see Section 3).

For hBRR2^T1^–hJab1^ΔC^ complex crystals, soaking was used as a strategy to obtain additional fragment-bound structures. Preformed hBRR2^T1^–hJab1^ΔC^ crystals were incubated with small molecules at final concentrations of 50 m*M* (compounds **26** and **39**) or 100 m*M* (compounds **18**, **34**, **50**, **76**, **78**, **86** and **24**) in reservoir solution with 10%(*v*/*v*) DMSO for 1 h (compounds **18**, **26**, **34**, **39**, **50**, **76**, **78** and **86**) or for 5 min (compound **24**). The soaked crystals were cryoprotected by transfer to reservoir solution supplemented with 25%(*v*/*v*) ethylene glycol and were flash-cooled in liquid nitrogen for subsequent diffraction data collection.

Diffraction data were collected on beamline 14.1 of the BESSY II storage ring, Berlin, Germany and were processed with *XDS* (Kabsch, 2010 ▸). Molecular replacement with *Phaser* (McCoy *et al.*, 2007 ▸) was performed in *Phenix* (Liebschner *et al.*, 2019 ▸). Structures were refined by manual model building with *Coot* (Emsley *et al.*, 2010 ▸) and automated refinement with *phenix.refine* (Afonine *et al.*, 2012 ▸). The ligands were manually positioned into the densities, and parameters for the ligands were derived from the *eLBOW* tool of *Phenix* (Moriarty *et al.*, 2009 ▸). In the final rounds of refinement, the *B* factors of the protein were refined by translation–libration–screw rotation (TLS) refinement. For the structure of hBRR2^T1^ with sulfaguanidine, three TLS groups were defined, spanning residues 402–812, 813–1813 and 1814–2125 of hBRR2^T1^. For the structures of hBRR2^T1^–hJab1^ΔC^ in complex with compounds, one TLS group was defined for hBRR2^T1^ and a second TLS group for hJab1^ΔC^. The *B* factors of compounds, water molecules and ethylene glycol molecules were refined isotropically. In the course of the refinement, the occupancy of the compounds was set to 1.0. Model quality was assessed with *MolProbity* (Chen *et al.*, 2010 ▸; Williams *et al.*, 2018 ▸). 3D structure figures were prepared with *PyMOL* (Schrödinger). Binding schematics were prepared with *LigPlot* (Wallace *et al.*, 1995 ▸). Crystallographic parameters are provided in Table 1 ▸.

### 2.3. Docking and analogue search

Template-guided docking was carried out with *FlexX* (Rarey *et al.*, 1996 ▸; Metz *et al.*, 2021 ▸) using the crystallographic binding pose of sulfaguanidine or a substructure as the template. The *FlexX* algorithm deconstructs every docked compound into smaller substructures, aligns the best matching substructures to the corresponding substructures of the template and reconstructs the ligand while allowing it to flexibly explore the environment of the binding pocket. Searching for suitable analogues that contain sulfaguanidine as a substructure yielded 52 analogues from MolPort and 315 from the National Cancer Institute Developmental Thera­peutics Program (NCI/DTP; https://dtp.cancer.gov). Searches were conducted using the MolPort Chemical Search node (SIA MolPort, Latvia) within *Konstanz Information Miner* (*KNIME*) version 3.4.0 (Berthold *et al.*, 2008 ▸; Kim *et al.*, 2016 ▸). To increase the number of suitable candidates, we included all tautomeric and protonation states of the interacting sulfaguanidine core motif [*N*-(aminomethyl)methanesulfonamide and *N*′-methylsulfonylmethanimidamide] as query structures, resulting in the retrieval of 187 and 22 additional compounds from MolPort and NCI/DTP, respectively. However, we noticed that the MolPort web interface still returned additional results compared with the *KNIME* node, presumably due to an internal normalization of the query structures provided as SMILES strings in *KNIME*. Extending the comprehensive search to the MolPort web interface yielded 396 suitable analogues from MolPort.

To facilitate template-guided docking of additional ana­logues that contain the core motif but not the complete sulfaguanidine moiety as a substructure, we used six sub­structures of sulfaguanidine in its crystallographically observed pose (Figs. 1 ▸ *b* and 1 ▸ *c*; Supplementary Fig. S1) as templates. Only the three best-scoring docking poses of each compound were retained, resulting in the expected overlap of the docked analogues with the crystallographic sulfaguanidine pose for all possible cases. Compounds that could not be docked due to an unavoidable steric overlap with the protein did not result in a docking pose.

To enrich small but efficiently binding ligands, all docked poses were then rescored with *DrugScore DSX* (Neudert & Klebe, 2011 ▸) using default settings and were ranked by the DSX score normalized by the number of non-H atoms. All poses were visually assessed by visualization with the *PyMOL* session produced by *DSX*, highlighting interactions and the contribution of each atom to the total DSX score.

The primary aim of the above candidate selection was to identify extended sulfaguanidine analogues that form additional interactions and may exhibit enhanced affinities. However, due to the limited pool of selected compounds, we included additional compounds that (i) are of a similar size to or even smaller than sulfaguanidine yet may elucidate which interactions are crucial for or may improve the binding pose of sulfaguanidine, (ii) extend from the sulfaguanidine pose but do not form optimal interactions in the docking pose yet may do so upon induced-fit adjustment of the binding site that was not considered during docking or (iii) contain chemical motifs that are potentially unsuited for testing in functional assays due to chemical reactivity or unspecific activity, for example pan-assay interference compounds (Baell & Nissink, 2018 ▸) or imines, yet may elucidate potential interactions if bound in a crystal structure. The selected compounds are listed in Supplementary Table S1.

### 2.4. Small-molecule stocks

Small molecules were obtained from NCI/DTP (https://dtp.cancer.gov), purchased from MolPort or collected from an in-house fragment library stock. All substances were dissolved in 100% DMSO at concentrations ranging from 250 m*M* to 1 *M* depending on their solubility. Small-molecule stocks were stored at −20°C until further use.

### 2.5. RNA duplex-unwinding assays

RNA duplex-unwinding assays were performed with a U4*/U6 di-snRNA substrate (U4*, [^32^P]-labelled U4 snRNA) that was prepared as described previously (Theuser *et al.*, 2016 ▸). 200 n*M* hBRR2^T1^ was incubated with or without compounds [at a concentration of 1 m*M* in the initial screen and at the indicated concentrations in IC_50_ evaluation assays; final DMSO concentration of 4 or 10%(*v*/*v*)] for 3 min at 30°C in unwinding buffer [40 m*M* Tris–HCl pH 7.5, 50 m*M* NaCl, 0.5 m*M* MgCl_2_, 15 ng µl^−1^ acetylated BSA, 1 U µl^−1^ RNasin (Molox), 8%(*v*/*v*) glycerol]. Reactions were started by the addition of 1.7 m*M* ATP/MgCl_2_ and 10 µl samples were taken at defined time points and mixed in a 1:1 ratio with 40 m*M* Tris–HCl pH 7.4, 50 m*M* NaCl, 25 m*M* EDTA, 1%(*w*/*v*) SDS, 10%(*v*/*v*) glycerol, 0.05%(*w*/*v*) xylene cyanol, 0.05%(*w*/*v*) bromophenol blue. The samples were loaded onto 4% native PAGE. After electrophoresis at 170 V and 4°C for 2–3 h, the gels were transferred to a filter paper, dried and used for the exposure of a storage phosphor screen. Detection of radioactive bands was performed by scanning with a Typhoon phosphorimager (GE Healthcare).

## 3. Results

### 3.1. Initial fragment hit and identification of derivatives

In this study, we worked with an N-terminally truncated version of the human spliceosomal RNA helicase, hBRR2, which lacks the auto-inhibitory N-terminal region of the enzyme (residues 395–2129; hBRR2^T1^) and exhibits enhanced RNA helicase activity compared with the full-length protein (Santos *et al.*, 2012 ▸). We identified sulfaguanidine [4-amino-*N*-(aminoiminomethyl)-benzolsulfonamide], a known antibacterial drug (Abidi *et al.*, 2018 ▸), as a ligand of hBRR2^T1^ in a crystallization screen with additives (Silver Bullets condition 12; Hampton Research, catalogue No. HR2-096). In this hBRR2^T1^ crystal structure, sulfaguanidine is positioned at the interface between the two helicase cassettes, directly contacting the N-terminal HB and IG domains as well as the C-terminal RecA2 domain (Figs. 1 ▸ *a*–1 ▸ *c*). The binding involves multiple hydrogen bonds, such as those between the main chain of Tyr1238 of the N-terminal IG domain and Gln1707 of the C-terminal RecA-2 domain and the sulfonyl O atom (Fig. 1 ▸ *c*). The *para*-positioned amino group forms a hydrogen bond to Glu1097 of the HB domain and the guanidine moiety is contacted by a hydrogen bond to the main chain of His1236 of the IG domain. Furthermore, the binding is supported by π-stacking interactions with Tyr1238 and van der Waals interactions of the benzene ring with Trp1222 (N-terminal IG domain) and Asn1531 (N-terminal RecA2 domain; Fig. 1 ▸ *c*). Sulfaguanidine is contacted by a multitude of inter­actions including domains of both helicase cassettes.

It has been shown that only the N-terminal cassette (NC) of BRR2 is an active helicase, whereas the C-terminal cassette (CC) is inactive in unwinding and ATP hydrolysis (Kim & Rossi, 1999 ▸; Santos *et al.*, 2012 ▸). The CC is assumed to regulate the activity of the NC via the interface between the two cassettes, and it has been shown that residue exchanges that affect this interface influence the activity of the NC (Santos *et al.*, 2012 ▸; Vester *et al.*, 2020 ▸). Inhibitors of hBRR2 have recently been reported which bind at the interface between the helicase cassettes (Iwatani-Yoshihara *et al.*, 2017 ▸; Ito *et al.*, 2017 ▸). Sulfaguanidine occupies a different binding site displaced by about 9 Å from BRR2-inhibitory compounds (Supplementary Fig. S2) and did not influence the RNA helicase activity of hBRR2^T1^ (Supplementary Fig. S3). However, as sulfaguanidine qualifies as a fragment (molecular weight 214.2 g mol^−1^) and exhibits low lipophilicity (*c*log*P* = −1.1; Enhanced NCI Database Browser 2.2), further modification may be possible to yield hBRR2-modulating substances. We therefore set out to explore whether sulfaguanidine derivatives can be identified which exhibit altered binding and possibly hBRR2-modulatory activities.

Based on the structure of the hBRR2^T1^–sulfaguanidine complex, we carried out structure-guided docking of sulfaguanidine derivatives. Sulfaguanidine as well as six substructures of sulfaguanidine in its crystallographically observed pose (Supplementary Fig. S1) were used as templates for guided docking with the aim of identifying molecules that might exhibit a similar binding mode. 93 compounds with favourable docking poses were ordered from the NCI/DTP, purchased from MolPort or collected from an in-house fragment library stock (Supplementary Table S1; Supplementary Fig. S4). We attempted to co-crystallize hBRR2^T1^ with the derivatives under conditions that yielded hBRR2^T1^–sulfaguanidine crystals or to soak derivatives into preformed hBRR2^T1^ crystals. However, neither strategy yielded hBRR2^T1^-derivative structures, possibly because crystal formation was impaired by the high fragment concentrations in some co-crystallization trials or because the high-salt crystallization conditions (including 1.5 *M* malonate) may have counteracted fragment binding during co-crystallization or soaking. Crystal soaking was also hampered by the deterioration of hBRR2^T1^ crystals upon soaking with DMSO-containing solutions.

### 3.2. Binding of sulfaguanidine derivatives to conformationally rearranged hBRR2^T1^

hBRR2 or hBRR2^T1^ can bind to the C-terminal Jab1/MPN domain (residues 2064–2335) of the hPRPF8 protein (Mozaffari-Jovin *et al.*, 2013 ▸; Maeder *et al.*, 2009 ▸). When the C-terminal tail of the hPRPF8 Jab1/MPN domain extends into the RNA-binding tunnel of hBRR2, the domain acts as an inhibitor of the helicase; when the tail is removed, the hPRPF8 Jab1/MPN domain activates hBRR2 helicase activity, an effect that can be mimicked by a C-terminally truncated version of the domain (residues 2064–2320; hJab1^ΔC^; Mozaffari-Jovin *et al.*, 2013 ▸). We have recently reported a crystal structure of a hBRR2^T1^–hJab1^ΔC^ complex in which the hBRR2^T1^ helicase cassettes adopted a different relative orientation compared with that in isolated hBRR2^T1^ (Fig. 1 ▸ *d*; PDB entry 6s8q; Vester *et al.*, 2020 ▸). In particular, the sulfaguanidine-binding surfaces of hBRR2^T1^ are rearranged in the hBRR2^T1^–hJab1^ΔC^ crystals, with increased distances between residues of the two cassettes that contact sulfaguanidine in the hBRR2^T1^–sulfaguanidine structure (Fig. 1 ▸ *e*). In the following, the conformation of hBRR2 in the monomeric hBRR2^T1^ crystal structure will be referred to as conformation 1, whereas the conformation of hBrr2 in the hBRR2^T1^–hJab1^ΔC^ complex crystals will be referred to as conformation 2.

As the crystallization condition of the hBRR2^T1^–hJab1^ΔC^ complex [0.1 *M* HEPES–NaOH pH 8.0, 0.1 *M* MgCl_2_, 8%(*w*/*v*) PEG 3350] appeared to be more suitable for fragment binding, and as hBRR2^T1^–hJab1^ΔC^ crystals grown under these conditions were stable in solutions of 10%(*v*/*v*) DMSO, we decided to explore whether, and if so how, sulfaguanidine and derivatives might bind to the hBRR2^T1^–hJab1^ΔC^ complex with rearranged helicase cassettes. To this end, we selected sulfaguanidine and a subset of 55 compounds from our collection of sulfaguanidine derivatives, which was enriched in higher structural similarity to sulfaguanidine, for crystallo­graphic binding studies using the hBRR2^T1^–hJab1^ΔC^ crystals. Sulfaguanidine itself did not bind to the hBRR2^T1^–hJab1^ΔC^ crystals. However, crystals soaked with nine derivatives yielded electron densities indicating clear binding of the substances (Supplementary Fig. S5; crystallographic parameters are listed in Table 1 ▸).

**Figure 2:**
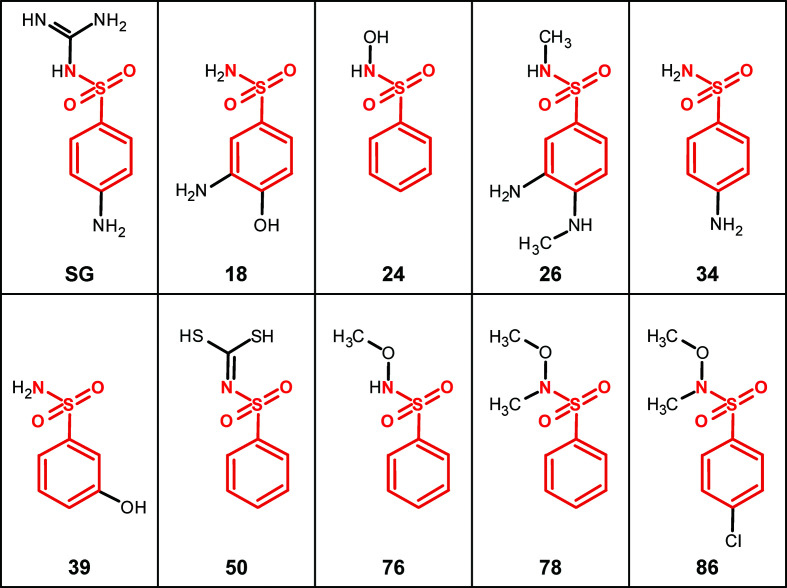
Fragment hits. Chemical structures of the fragments are shown with their shared substructure highlighted in red. SG, sulfaguanidine; numbers, fragments.

**Figure 3:**
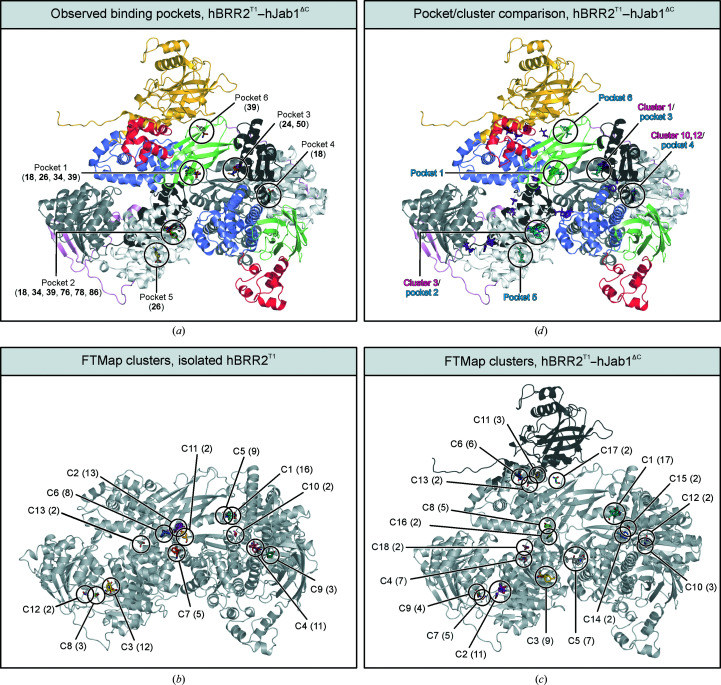
(*a*) The binding sites of nine fragments on the hBRR2^T1^–hJab1^ΔC^ complex. The crystal structures of the fragment complexes were aligned with the crystal structure of unliganded hBRR2^T1^–hJab1^ΔC^ (PDB entry 6s8q), and only the unliganded hBRR2^T1^–hJab1^ΔC^ structure is shown as a cartoon for clarity. Pocket 1 corresponds to part of the sulfaguanidine binding site in isolated hBRR2^T1^ and is bound by fragments **18**, **26**, **34** and **39**. Pocket 2 is located in the N-­terminal RecA1 domain and is bound by fragments **18**, **34** and **39** (pose 1) and fragments **76**, **78** and **86** (pose 2). Pocket 3 is located at the interface between the cassettes at a distance of approximately 21 Å from pocket 1 and is bound by fragments **24** and **50**. Pocket 4 corresponds to the ATP-binding pocket of the CC and is bound by fragment **18**. Pocket 5 is located on the surface of hBRR2^T1^ at the N-terminal RecA1 domain and is bound by compound **26**. Pocket 6 is positioned at the interface between the N-terminal IG domain of hBRR2^T1^ and hJab1^ΔC^ and is bound by fragment **39**. Fragments are shown as sticks and coloured by atom type as in Fig. 1 ▸(*c*), except that fragment C atoms are coloured by fragment. (*b*) *FTMap* results for isolated hBRR2^T1^ (PDB entry 4f91; grey cartoon). The *FTMap* server was used to predict the binding of probes to the crystal structure of isolated hBRR2^T1^. Probes that bind to the same site form a cluster (C1–C13, clusters 1–13). The clusters are ranked according to the number of predicted interacting probe molecules, with lower cluster numbers corresponding to a larger number of probes. Numbers of interacting probes are indicated in parentheses. Predicted interacting probe molecules are shown as sticks and coloured by atom type as in Fig. 1 ▸(*c*), except that probe molecule C atoms are coloured by probe molecule. (*c*) *FTMap* results for the hBRR2^T1^–hJab1^ΔC^ complex (PDB entry 6s8q; hBRR2^T1^, grey cartoon; hJab1^ΔC^, dark grey cartoon). Clusters and bound probes are shown and labelled as in (*b*). (*d*) Comparison of predicted hot spots and identified fragment-binding pockets. The structures of all hBRR2^T1^–hJab1^ΔC^–fragment complexes were aligned with the unliganded hBRR2^T1^–hJab1^ΔC^ complex structure including the *FTMap*-predicted clusters. Interacting fragments and *FTMap*-predicted interacting probe molecules are shown as sticks. Interacting fragments, cyan; *FTMap*-predicted interacting probe molecules, magenta. Fragment-binding pockets are labelled as in (*a*) and clusters are labelled as in (*c*).

All bound compounds exhibited the same 4-aminophenyl sulfonamide scaffold (Fig. 2 ▸). A total of six pockets were identified as binding sites of the ligands (Fig. 3 ▸ *a*). Five compounds (**24**, **50**, **76**, **78** and **86**) bound only to a single pocket, two fragments (**26** and **34**) bound at two pockets and two substances (**18** and **39**) bound at three pockets simultaneously. Density for an additional molecule of compound **18** bound at two neighbouring hBRR2^T1^–hJab1^ΔC^ complexes in the crystal lattice emerged during later stages of refinement; as it depended on the crystal packing, this binding site was excluded from further analysis.

Four of the compounds (**18**, **26**, **34** and **39**) bound at the cassette interface regions of the original sulfaguanidine binding site of hBRR2^T1^ (referred to as pocket 1; Fig. 3 ▸ *a*). All four fragments that occupied pocket 1 exhibited a similar binding pose as sulfaguanidine in the hBRR2^T1^ structure (Fig. 3 ▸ *a*). Six fragments bound additionally (**18**, **34** and **39**) or alternatively (**76**, **78** and **86**) at pocket 2, located within the N-terminal RecA1 domain, with two different binding modes (binding mode 1 for compounds **18**, **34** and **39**; binding mode 2 for compounds **76**, **78** and **86**; Fig. 3 ▸ *a*). Two fragments, compounds **24** and **50**, bound exclusively in pocket 3 located at the interface between the helicase cassettes and including residues from the N-terminal IG domain and the C-terminal RecA2 and WH domains, albeit with a distance of approximately 21 Å to pocket 1 (Fig. 3 ▸ *a*). Compounds **24** and **50** again exhibited different binding poses. Compound **18** exhibited a third binding site, pocket 4, corresponding to the C-terminal ATP-binding site of hBRR2^T1^ (Fig. 3 ▸ *a*). Pocket 5 is located on the surface of the N-terminal RecA1 domain and was bound by compound **26** (Fig. 3 ▸ *a*). Finally, compound **39** not only bound to pockets 1 and 2, but also occupied pocket 6 between the N-terminal IG domain of hBRR2^T1^ and hJab1^ΔC^ (Fig. 3 ▸ *a*).

The lack of binding of sulfaguanidine in the hBRR2^T1^–hJab1^ΔC^ complex structure (conformation 2) is most likely due to the altered binding site in this complex compared with the isolated hBRR2^T1^ structure (conformation 1; Fig. 1 ▸ *e*), which results in decreased affinity of the compound. Therefore, this finding suggests that the derivatives that did bind at pocket 1 exhibit improved binding to the respective part of the pocket compared with sulfaguanidine. These observations show that some of the derivatives exhibit sufficient complementarity to parts of the original sulfaguanidine binding site to remain associated with regions of the original sulfaguanidine binding site upon conformational rearrangement. Remarkably, however, the nine derivatives also bound at entirely new binding pockets and adopted diverse binding poses in these pockets.

### 3.3. Conformation-dependent binding hot spots in hBRR2^T1^

It has been suggested that the tendency of fragment deriv­atives to retain the binding mode of the original hit depends on whether the binding site represents a binding hot spot (Kozakov, Hall, Jehle *et al.*, 2015 ▸). Hot spots are regions of a protein that contribute a substantial fraction of binding free energy upon interaction with a ligand and thus can offer a binding site to diverse fragments (Hall *et al.*, 2015 ▸). Hot spots have been experimentally delineated, for example, via the identification of hot spot residues by alanine-scanning mutagenesis (DeLano, 2002 ▸) or by determining crystal structures after soaking with organic solvents (Mattos & Ringe, 1996 ▸). In good agreement with experimental data, hot spots can also be predicted with the *FTMap* server (Vajda *et al.*, 2015 ▸), which calculates the interaction energy of 16 probe molecules upon binding protein surface areas (Kozakov, Grove *et al.*, 2015 ▸). Probe molecules that bind to overlapping sites constitute a cluster, which identifies a hot spot. The clusters are ranked by the number of interacting probe molecules (Hall *et al.*, 2015 ▸).

We used the *FTMap* server to predict hot spots on hBRR2^T1^ in the two conformations observed in the isolated hBRR2^T1^ and the hBRR2^T1^–hJab1^ΔC^ crystal structures (Figs. 3 ▸ *b* and 3 ▸ *c*). 13 hot spots were predicted for isolated hBRR2^T1^ (Fig. 3 ▸ *b*). The primary hot spot (16 interacting probes) is located at the interface between the cassettes, coinciding with the position of known hBRR2 inhibitors (Iwatani-Yoshihara *et al.*, 2017 ▸; Ito *et al.*, 2017 ▸). A second hot spot (13 interacting probes) overlaps with the original sulfaguanidine-binding site (Fig. 3 ▸ *b*). This hot spot is surrounded by three other hot spots (ranks 6, 7 and 11). Additional hot spots were predicted near the ATP-binding pockets of the NC (rank 3) and CC (rank 4), and the remaining hot spots are distributed across hBRR2^T1^ (Fig. 3 ▸ *b*).

The nature and ranks of the hot spots predicted for hBRR2^T1^ in conformation 2 observed in the hBRR2^T1^–hJab1^ΔC^ complex differed markedly (Fig. 3 ▸ *c*). While the primary hot spot was again predicted at the cassette interface, it overlapped with the binding sites of compounds **24** and **50** at pocket 3 (Figs. 3 ▸ *c* and 3 ▸ *d*). Regions corresponding to the original sulfaguanidine binding site in conformation 1 were not predicted to be hot spots in conformation 2 of the hBRR2^T1^–hJab1^ΔC^ complex. Instead, the N-terminal nucleotide-binding pocket is the second ranked predicted hot spot (Fig. 3 ▸ *c*). A new predicted hot spot (rank 3, nine interacting probes) is located in the N-terminal RecA1 domain (Fig. 3 ▸ *c*), corresponding to binding pocket 2 (Fig. 3 ▸ *d*) that we identified as the binding site for six fragments (Fig. 3 ▸ *a*). However, while four additional hot spots were predicted at the interface of hBRR2^T1^ and hJab1^ΔC^, these sites differed from pocket 6 at this interface, which we observed as a binding site for compound **39** (Figs. 3 ▸ *c* and 3 ▸ *d*). Omitting the hJab1^ΔC^ structural coordinates during the *FTMap* server analysis did not lead to major differences in the predicted hot spots, suggesting that hot spots on the surface of hBRR2^T1^ predominantly depend on the conformation of hBRR2^T1^ rather than the presence of hJab1^ΔC^. Overall, this analysis suggests that predicted hot spots can change decis­ively when a protein undergoes conformational changes, even if the rearrangement preserves the structures of individual domains and mainly constitutes rigid-body rearrangements of domain assemblies (in this case the hBRR2 helicase cassettes). Furthermore, in the case studied here, the predicted hot spots overlap with, but are not perfectly congruent with, observed fragment-binding sites.

### 3.4. Weak interactions favour low binding-mode conservation

We did not find that the preferences of ligands for a specific binding pocket correlated with a trend in general physicochemical properties such as molecular mass, lipophilicity (log*P* value) or the number of rotatable bonds (Table 2 ▸). Therefore, we inspected the chemical details of the interactions within the pockets, which might hint at features of the ligands that explain their binding preferences.

**Figure 4:**
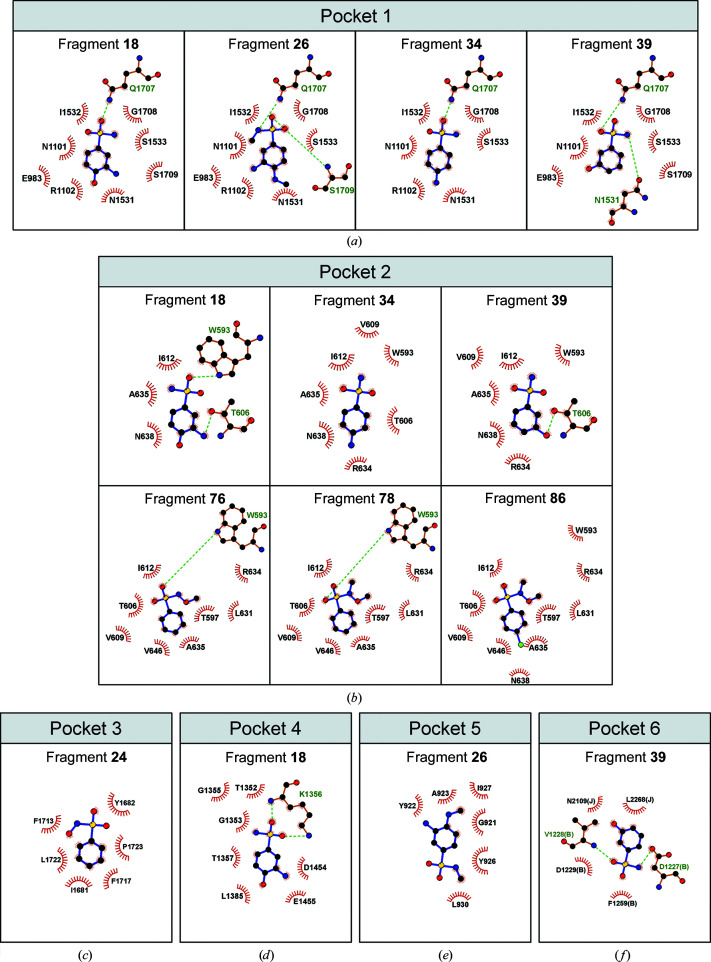
*LigPlot* (Wallace *et al.*, 1995 ▸) representation of the fragment-binding modes in the pockets of hBRR2^T1^ in the hBRR2^T1^–hJab1^ΔC^ complex. For the location of the pockets, see Fig. 3 ▸(*a*). Residues of hBRR2^T1^ involved in interactions with the fragments are labelled. Red rays, van der Waals interactions; green dashes, hydrogen bonds. (*a*) Interactions of fragments **18**, **26**, **34** and **39** in pocket 1. (*b*) Interactions of fragments **18**, **34** and **39** (pose 1) and **76**, **78** and **86** (pose 2) in pocket 2. (*c*) Interactions of fragment **24** in pocket 3. (*d*) Interactions of fragment **18** in pocket 4 (C-terminal ATP-binding site). (*e*) Interactions of fragment **26** in pocket 5 (surface of the N-terminal RecA1 domain). (*f*) Interactions of fragment **39** in pocket 6 (interface between hBRR2^T1^ and hJab1^ΔC^); hBRR2^T1^ and hJab1^ΔC^ residues are labelled ‘B’ and ‘J’ in parentheses, respectively.

Pocket 1, corresponding to the altered sulfaguanidine binding site, was occupied by fragments **18**, **26**, **34** and **39**. Pocket 1 was not a predicted hot spot in this conformation 2. Compounds bound at this position are likely to exhibit some conserved interactions compared with the original sulfa­guanidine binding mode. Indeed, some key interactions with residues from the C-terminal cassette remained (Fig. 4 ▸ *a*). Among them are van der Waals interactions with Asn1531, Gly1708 and Ser1709 and a hydrogen bond to Gln1707. Nevertheless, several of the hydrogen bonds of the original sulfaguanidine binding mode are missing and the compounds predominantly exhibit rather weak hydrophobic contacts.

The ligands of pocket 2, which is located within the N-terminal RecA1 domain, exhibited two different binding poses. One pose is adopted by fragments **18**, **34** and **39** and the other is adopted by fragments **76**, **78** and **86** (Fig. 4 ▸ *b*). The main conserved property of ligands **76**, **78** and **86** compared with ligands **18**, **34** and **39** is an additional methoxy group attached to the amino group. The van der Waals interactions of the methoxy group with Leu631, Thr597 and Arg634 are probably responsible for the alternative pose of ligands **76**, **78** and **86** compared with ligands **18**, **34** and **39** at pocket 2.

Ligands **24** and **50** bound exclusively at pocket 3, which is positioned at the interface between the N-terminal IG domain and the C-terminal RecA2/WH domains. Fragment **24** also engages in a few hydrophobic interactions (Fig. 4 ▸ *c*; the inter­actions of fragment **50** are not shown because of the relatively low resolution, 3.5 Å, of the corresponding complex structure).

Pockets 4, 5 and 6 were each only bound by one fragment. The position of compound **18** in pocket 4 directly overlaps with the observed position of ATP in the C-terminal ATP-binding site (Fig. 4 ▸ *d*). Indeed, the sulfonyl group of compound **18** established interactions with key residues of ATP-binding motifs. A hydrogen bond is formed to Lys1356 of motif I (which usually contacts the β- and γ-phosphate of ATP), as well as van der Waals interactions with Gly1353, Gly1355 (part of the flexible phosphate-binding loop) and Asp1454 and Glu1455 of motif II (responsible for the coordination of magnesium and, in active helicase modules, for the activation of a water molecule for hydrolysis). Perhaps surprisingly, we did not detect binding of compound **18** to the N-terminal ATP-binding pocket. The C-terminal helicase cassette is inactive in ATP hydrolysis, the motifs deviate from those of the N-terminal cassette (Santos *et al.*, 2012 ▸; Kim & Rossi, 1999 ▸) and the pocket of the N-terminal cassette is probably more flexible to undergo frequent ATP hydrolysis cycles (Absmeier *et al.*, 2021 ▸); this probably contributes to the lack of binding of compound **18** at the N-terminal site.

Compound **26** bound in a groove located on the surface of the N-terminal RecA1 domain of hBRR2^T1^: pocket 5 (Fig. 4 ▸ *e*). In this position, the *para*-positioned methyl group of compound **26** is involved in multiple van der Waals inter­actions with Gly921, Tyr922, Ala923 and Ile927.

Finally, pocket 6 was occupied by compound **39**, which additionally bound at pocket 1 and pocket 2. Pocket 6 is located between hBRR2^T1^ and the interaction partner hJab1^ΔC^ (Fig. 4 ▸ *f*). Here, compound **39** sustains hydrogen bonds to Asp1227 and Val1228 and van der Waals interactions with Asp1229 and Phe1259 of hBRR2^T1^ and with Leu2268 and Asn2109 of hJab1^ΔC^.

In conclusion, all ligands bound to hBRR2^T1^ in conformation 2 appeared to be contacted mainly through van der Waals interactions, often mediated by the core benzene ring, whereas stronger types of interaction are not frequently observed. These comparably weak interactions are likely to give rise to a low binding affinity of the compounds in the respective pocket and therefore facilitate changes in binding modes.

### 3.5. Some fragments inhibit BRR2 helicase activity

To further test whether the bound fragments identified in our crystallographic screen might constitute interesting hits for the development of new hBRR2-modulating substances, we tested the effects of the fragments identified in co-crystal structures on hBRR2 helicase activity. hBRR2^T1^-mediated RNA unwinding in the presence of compounds compared with a DMSO control was monitored by single time-point unwinding assays of a U4/U6 di-snRNA duplex, in which the U4 snRNA strand was radioactively labelled. As a positive control for a substance with known effects on unwinding activity, we used a previously published hBRR2 inhibitor: compound **33a** developed by Ito *et al.* (2017 ▸).

While compound **33a** efficiently inhibited hBRR2^T1^-mediated U4/U6 unwinding (45% of the unwinding amplitude of the DMSO control after 2 min), we did not detect any effect of sulfaguanidine on unwinding at 300 µ*M* or 1 m*M* (Supplementary Fig. S3).

Of the nine interacting derivatives (compounds **18**, **24**, **26**, **34**, **39**, **50**, **76**, **78** and **86**), we observed reduced unwinding by hBRR2^T1^ upon the addition of compounds **24** and **50**, whereas the other compounds had either no or only a marginal effect (Supplementary Fig. S6*a* ). The IC_50_ values of compounds **24** and **50** were >90 µ*M* and >1 m*M* according to concentration-dependent assays (Supplementary Figs. S6*b* and S6*c* ).

In conclusion, only compounds **24** and **50** clearly bound to hBRR2^T1^ and additionally showed an effect on helicase activity. They also both bound to the same pocket 3. Compound **24** is known as Piloty’s acid, which can produce nitroxyl and nitrous oxide (Smulik-Izydorczyk *et al.*, 2019 ▸) and is a known inhibitor of aldehyde dehydrogenase (Nagasawa *et al.*, 1995 ▸). However, our assays were conducted at a pH below 8.0, where such a decomposition of compound **24** is unlikely. An effect of both compounds **24** and **50** upon the binding of pocket 3 would be in line with the effect of the known inhibitors of hBRR2 that also bind in this region (Iwatani-Yoshihara *et al.*, 2017 ▸; Ito *et al.*, 2017 ▸). However, we cannot exclude any nonspecific effects of these compounds at the high concentrations used in biochemical assays. Further elaboration of these fragments would be required to convert them to compounds with higher potency and specificity.

## 4. Discussion

In this fragment-screening approach for the spliceosomal helicase hBRR2, multiple binding sites and binding poses were identified for ten fragments with a conserved chemical core structure. Based on the initial hit sulfaguanidine as a ligand at the interface of the helicase cassettes, nine related fragments were identified to bind to six different pockets. We subsequently identified protein and ligand properties as a basis for the drastic alterations in binding site and binding pose.

The first obvious cause for changed or abrogated binding are the conformational differences of hBRR2^T1^ in the two different crystal forms (isolated hBrr2^T1^ versus the hBRR2^T1^–hJab1^ΔC^ complex). In particular, the position of the CC relative to the NC is shifted in the hBRR2^T1^–hJab1^ΔC^ complex structure, so that the interface between the helicase cassettes, and thus the sulfaguanidine binding site, is affected (Fig. 1 ▸ *e*). Therefore, it is not surprising that sulfaguanidine as well as several compounds with the same chemical core structure no longer bound to the remaining parts of the original pocket 1 in the hBRR2^T1^–hJab1^ΔC^ complex crystals. Surprisingly, however, five other pockets were occupied by sulfaguanidine-derived compounds. Although the conformational differences between isolated hBRR2^T1^ and the hBRR2^T1^–hJab1^ΔC^ complex strongly affect the sulfaguanidine-binding pocket 1 [root-mean-square deviation (r.m.s.d.) of 4.12 Å upon alignment of domains from pocket 1 in PDB entries 4f91 and 6s8q], the other pockets remain essentially unaltered (r.m.s.d. values of around 0.44–0.73 Å upon the alignment of the domains contributing to the pockets; Supplementary Table S2).

Previously, Kozakov and coworkers hypothesized that the hot spots of a protein can determine the binding potential and therefore the degree of binding-mode conservation for ligands (Kozakov, Hall, Jehle *et al.*, 2015 ▸). The *FTMap* analyser of Kozakov, Grove *et al.* (2015 ▸) predicted drastically different hot spots for hBRR2^T1^ in the two crystalline conformations. The cluster/hot spot of the sulfaguanidine binding site was predicted for hBRR2^T1^ in the isolated conformation but not in the hJab1^ΔC^ complex conformation. In agreement with the altered binding site and the absence of a predicted hot spot, only four of the compounds bound at parts of pocket 1 in conformation 2. Remarkably, the *FTMap* server predicted a new hot spot, cluster 3, which was only present in the hJab1^ΔC^ complex conformation (pocket 2; Fig. 3 ▸ *c*), consistent with the observed binding of six fragments (compounds **18**, **34**, **39**, **76**, **78** and **86**) to this pocket (Fig. 3 ▸ *d*). Other predicted clusters, 1, 10 and 12, were also occupied by tested fragments (pocket 3 by compounds **24** and **50** and pocket 4 by compound **18**; Figs. 3 ▸ *c* and 3 ▸ *d*). In conclusion, the predicted hot spots differ drastically in both conformations, although the rigid-body alterations only have a very minor structural impact on the majority of the protein pockets, with the exception of pocket 1. In agreement, some of the predicted hot spots are occupied by fragments from our screen.

The properties of the fragments from our screen, such as size, lipophilicity and the number of rotatable bonds (Table 2 ▸), are in the typical ranges for fragments used in screens (Congreve *et al.*, 2003 ▸). However, all nine of the bound fragments as well as the conserved core have a size of more than 150 Da. Drwal and coworkers previously noted, based on an analysis of PDB structures, that the binding mode of a fragment with molecular mass of 150 Da or above tends to be conserved (Drwal *et al.*, 2018 ▸). In contrast to this suggestion, we observed marked binding-site and binding-pose variations with related compounds above this size limit. Although our study solely resembles an exemplary test case of the binding of ten similar fragments to a protein, it demonstrates that these unexpected observations can be made in a reasonable approach.

Clearly, the lack of strong hydrogen-bond interactions is probably one of the main causes of low binding-mode conservation, as also noted in the study by Malhotra & Karanicolas (2017 ▸). The weak van der Waals interactions of the benzene ring and methyl groups are likely to give rise to low affinity of the compounds within each binding site of hBRR2^T1^. Further moieties of this core can promote preferential binding to other pockets, which may have an improved affinity for the differently decorated fragment derivative. A remarkable example from our screen is the binding mode of compound **24** compared with compound **76**. The compounds only differ in a methyl group, so that compound **24** contains a hydroxyl group that is linked to the amino group, whereas compound **76** has a methoxy group. This modification is sufficient to cause the binding of compound **24** to pocket 3 in the interface between the cassettes, whereas compound **76** is bound in pocket 2 within the N-terminal RecA1 domain.

The fragments identified as ligands in our screen could be used for further elaboration into more potent hBRR2 modulators. Due to its essential role in pre-mRNA splicing and due to the association of mutations that affect hBRR2 with the disease retinitis pigmentosa, this helicase is an interesting target for drug discovery. Fragments such as compounds **24** and **50** with initial effects on hBRR2-dependent RNA unwinding or fragment **39** within an interesting pocket at the hBRR2–hJab1^ΔC^ interface could be starting points for such approaches.

In general, hot spots are considered to be rather insensitive to the flexibility of a protein, in contrast to, for example, the shapes of binding sites (Kozakov *et al.*, 2011 ▸; Kozakov, Hall, Napoleon *et al.*, 2015 ▸). However, our analysis demonstrates that the conformation of a protein can drastically influence the location and the strength of hot spots. Whereas slight side-chain variations and a certain pocket flexibility are not decisive in most cases (Kozakov, Hall, Napoleon *et al.*, 2015 ▸), hot-spot predictions for proteins with high intrinsic flexibility should therefore take into account the known spectrum of structural variability. Indeed, conformational dynamics can be critical for the druggability of a protein and some potential binding sites might only be detected in nonstatic representations such as molecular-dynamics simulations, as demonstrated, for instance, for Bcl-xL (Brown & Hajduk, 2006 ▸). Also, in the case of CDK2 it was shown that the binding mode of known inhibitors differed drastically between two crystal forms: the monomeric inactive conformation of CDK2 and the active CDK2–cyclin complex form (Kontopidis *et al.*, 2006 ▸). Some inhibitors of CDK2 bound more strongly to the active complex, while others preferentially bound to the inactive monomeric form, so that structure–activity relationship data derived exclusively from one of the two forms could be misleading (Kontopidis *et al.*, 2006 ▸).

Clearly, only hot spots in physiologically relevant conformations are important and it has to be considered for FBDD that crystal structures may exhibit irrelevant binding sites. Especially for methods that assess ligand binding in solution, differences in hot spots within different simultaneously present conformations of the target protein should be considered. Observed binding of derivatives could potentially be related to changes in binding modes, which should be carefully validated by structural techniques at each step of fragment elaboration into a lead. Nevertheless, new binding modes might enable to explore the chemical space in novel, previously unidentified directions.

## 5. Related literature

The following reference is cited in the supporting information for this article: Sander *et al.* (2015 ▸).

## Supplementary Material

PDB reference: human Brr2 helicase region, complex with C-tail-deleted Jab1 and compound **18**, 8bc8

PDB reference: complex with C-tail-deleted Jab1 and compound **24**, 8bc9

PDB reference: complex with C-tail-deleted Jab1 and compound **26**, 8bca

PDB reference: complex with C-tail-deleted Jab1 and compound **34**, 8bcb

PDB reference: complex with C-tail-deleted Jab1 and compound **39**, 8bcc

PDB reference: complex with C-tail-deleted Jab1 and compound **50**, 8bcd

PDB reference: complex with C-tail-deleted Jab1 and compound **76**, 8bce

PDB reference: complex with C-tail-deleted Jab1 and compound **78**, 8bcf

PDB reference: complex with C-tail-deleted Jab1 and compound **86**, 8bcg

PDB reference: complex with sulfaguanidine, 8bch

Supplementary Tables and Figures. DOI: 10.1107/S2059798323001778/ni5026sup1.pdf

## Figures and Tables

**Table 1:** Crystallographic data Values in parentheses are for the highest resolution shell.

Data set	Compound **18**	Compound **24**	Compound **26**	Compound **34**	Compound **39**	Compound **50**	Compound **76**	Compound **78**	Compound **86**	Sulfaguanidine
PDB entry	8bc8	8bc9	8bca	8bcb	8bcc	8bcd	8bce	8bcf	8bcg	8bch
Data collection										
Wavelength (Å)	1.0332	1.0332	1.0332	1.0332	1.0332	1.0332	0.9184	0.9184	0.9184	0.9184
Temperature (K)	100	100	100	100	100	100	100	100	100	100
Space group	*P*2_1_2_1_2_1_	*P*2_1_2_1_2_1_	*P*2_1_2_1_2_1_	*P*2_1_2_1_2_1_	*P*2_1_2_1_2_1_	*P*2_1_2_1_2_1_	*P*2_1_2_1_2_1_	*P*2_1_2_1_2_1_	*P*2_1_2_1_2_1_	*C*2
*a*, *b*, *c* (Å)	100.7, 119.2, 187.2	99.8, 119.2, 188.3	100.4, 119.4, 187.8	100.2, 119.2, 186.7	100.4, 188.2, 119.4	101.1, 121.9, 186.3	100.1, 119.1, 188.0	99.6, 118.8, 187.0	99.9, 118.9, 188.1	146.5, 149.8, 141.7
α, β, γ (°)	90, 90, 90	90, 90, 90	90, 90, 90	90, 90, 90	90, 90, 90	90, 90, 90	90, 90, 90	90, 90, 90	90, 90, 90	90, 120.3, 90
Resolution (Å)	50.00–2.39 (2.53–2.39)	50.00–2.30 (2.43–2.30)	50.00–2.80 (2.97–2.80)	50.00–2.38 (2.52–2.38)	50.00–2.35 (2.49–2.35)	50.00–3.50 (3.71–3.50)	50.00–2.05 (2.17–2.05)	50.00–2.42 (2.56–2.42)	50.00–2.39 (2.53–2.39)	50.00–2.87 (3.04–2.87)
Reflections										
Unique	89613 (14187)	100449 (15822)	56042 (8857)	89385 (13922)	94679 (14864)	29691 (4699)	139806 (21677)	85165 (13518)	89144 (14027)	58705 (9524)
Completeness (%)	99.7 (98.9)	199.6 (98.0)	99.4 (98.8)	98.7 (96.4)	99.6 (98.1)	99.6 (99.3)	99.2 (96.2)	99.9 (99.4)	99.7 (98.4)	97.1 (98.4)
Multiplicity	6.7 (6.8)	6.7 (6.4)	6.7 (6.8)	6.7 (6.4)	6.7 (6.5)	6.7 (6.9)	6.5 (5.6)	10.5 (10.5)	6.7 (6.9)	6.8 (6.9)
Data quality										
Intensity 〈*I*/σ(*I*)〉	9.57 (0.81)	14.75 (1.48)	8.05 (0.94)	9.20 (0.75)	8.55 (0.95)	8.10 (0.70)	13.63 (1.03)	9.14 (0.83)	8.68 (0.79)	10.2 (0.63)
*R*_meas_ † (%)	16.9 (253.9)	19.5 (123.8)	22.0 (207.4)	18.3 (266.2)	20.3 (227.1)	20.7 (286.5)	9.9 (163.4)	23.1 (263.6)	21.5 (262.1)	20.1 (327.2)
CC_1/2_ ‡	99.8 (28.9)	99.9 (57.0)	99.6 (37.4)	99.8 (24.2)	99.5 (33.1)	99.8 (30.4)	99.9 (41.9)	99.7 (36.5)	99.6 (28.5)	20.1 (327.2)
Wilson *B* value (Å^2^)	53.2	148.8	66.5	53.2	46.8	135.1	39.9	50.9	48.3	82.7
Refinement										
Resolution (Å)	50.00–2.39 (2.44–2.39)	50.00–2.30 (2.35–2.30)	50.00–2.80 (2.97–2.80)	50.00–2.38 (2.43–2.38)	50.00–2.35 (2.40–2.35)	50.00–3.50 (3.71–3.50)	50.00–2.05 (2.17–2.05)	50.00–2.42 (2.56–2.42)	50.00–2.39 (2.53–2.39)	50.00–2.87 (2.94–2.87)
Reflections										
Number	89597 (5607)	100439 (6220)	56033 (3446)	89373 (5537)	94667 (5848)	29675 (2514)	139459 (8335)	85152 (5389)	89138 (5499)	56190 (3286)
Test set (%)	2.34	2.09	3.75	2.35	2.22	5.0	1.50	2.47	2.36	3.58
*R*_work_ (%)	20.7 (32.8)	19.8 (28.4)	20.1 (35.7)	20.7 (34.6)	21.0 (33.2)	22.2 (37.6)	22.3 (41.3)	21.3 (33.3)	21.4 (35.00)	28.2 (60.6)
*R*_free_ (%)	24.5 (35.8)	24.9 (34.5)	25.9 (41.9)	25.8 (40.0)	25.8 (39.4)	28.0 (41.1)	26.8 (43.3)	27.5 (37.9)	26.9 (41.9)	33.7 (64.0)
Asymmetric unit										
Protein residues	1987	1987	1987	1984	1988	1985	1982	1987	1985	1722
Ethylene glycols	17	11	16	16	10	—	12	10	9	—
Compounds	3	1	2	2	3	1	1	1	1	1
Waters	1331	419	173	214	422	—	527	325	336	11
Mean temperature factors (Å^2^)										
All atoms	73.6	68.4	78.7	70.8	65.6	150.5	63.4	73.9	68.2	119.7
Macromolecules	4.1	68.8	79.1	71.1	66.1	151.2	64.2	74.3	68.6	119.8
Ligands	55.8	65.5	66.4	66.4	50.3	—	53.1	69.6	55.9	60.9
Compounds	4.2	53.1	55.2	55.2	49.5	117.5	44.5	57.2	55.5	59.9
Water molecules	52.4	53.4	54.9	50.4	49.8	—	48.1	52.8	49.8	53.6
R.m.s.d. from target geometry§										
Bond lengths (Å)	0.009	0.008	0.006	0.008	0.006	0.004	0.009	0.005	0.007	0.003
Bond angles (°)	1.072	0.917	0.779	0.985	0.801	0.690	0.979	0.752	0.885	0.610
Validation statistics										
Ramachandran plot, residues in										
Allowed regions (%)	4.2	3.1	3.7	3.2	2.7	4.7	3.7	3.1	4.3	5.6
Favoured regions (%)	95.5	96.6	96.0	96.5	97.0	94.9	97.0	96.7	95.6	94.3
Ramachandran plot *Z*-score (r.m.s.d.)										
Whole	−1.79 (0.17)	−1.39 (0.17)	−2.19 (0.16)	−1.93 (0.17)	−1.22 (0.17)	−1.95 (0.17)	−1.74 (0.17)	−1.42 (0.17)	−2.13 (0.16)	−2.58 (0.18)
Helix	−1.73 (0.14)	−1.35 (0.15)	−1.61 (0.14)	−1.59 (0.14)	−1.12 (0.15)	−1.37 (0.15)	−1.52 (0.15)	−1.03 (0.15)	−1.84 (0.14)	−1.84 (0.15)
Sheet	−0.14 (0.25)	0.27 (0.25)	−0.73 (0.24)	−0.15 (0.25)	0.27 (0.25)	−0.39 (0.26)	−0.12 (0.26)	−0.16 (0.25)	−0.17 (0.25)	−0.70 (0.29)
Loop	−0.78 (0.22)	−0.81 (0.21)	−1.39 (0.20)	−1.24 (0.21)	−0.77 (0.21)	−1.42 (0.21)	−0.98 (0.21)	−1.02 (0.21)	−1.30 (0.21)	−1.77 (0.22)
*MolProbity* clashscore¶ ††	9.49	7.64	9.81	7.36	7.42	12.36	9.00	8.42	8.06	12.70
*MolProbity* score¶	2.35	2.04	2.28	2.03	2.01	1.96	2.22	2.13	2.31	2.56
Poor rotamers (%)	3.1	3.4	4.6	3.3	3.8	0.0	4.0	4.1	5.6	5.5
C^β^ deviations (%)	0.0	0.0	0.0	0.0	0.0	0.0	0.0	0.0	0.0	0.0

† *R*_meas_(*I*) = 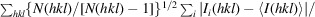 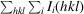 , where 〈*I*(*hkl*)〉 is the mean intensity of symmetry-equivalent reflections *hkl*, *I* *_i_* (*hkl*) is the intensity of a particular observation of *hkl* and *N*(*hkl*) is the number of redundant observations of reflection *hkl* (Diederichs & Karplus, 1997 ▸).

‡ CC_1/2_ = 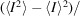 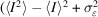 , where 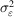 is the mean error within a half data set (Karplus & Diederichs, 2012 ▸).

§ Root-mean-square deviation.

¶ Calculated with *MolProbity* (Williams *et al.*, 2018 ▸).

†† Clashscore is the number of serious steric overlaps (>0.4) per 1000 atoms (Williams *et al.*, 2018 ▸).

**Table 2:** Properties of the fragment hits Properties are according to the NCI browser database (https://cactus.nci.nih.gov/ncidb2.2/).

Compound	Molecular mass (Da)	Hydrogen-bond acceptors/donors	Log*P*	Rotatable bonds
Sulfaguanidine	214.2	4/4	−1.07	3
Fragment **18**	188.2	5/3	−1.03	1
Fragment **20**	172.2	4/2	−0.55	1
Fragment **24**	173.2	4/2	0.25	2
Fragment **26**	215.3	5/3	−0.45	3
Fragment **34**	172.2	4/2	−0.55	1
Fragment **39**	173.2	4/3	−0.65	1
Fragment **50**	233.3	4/2	1.49	2
Fragment **76**	187.2	4/1	1.07	3
Fragment **78**	201.2	4/0	1.28	3
Fragment **86**	235.7	4/0	1.93	3
