# Supplementary material for: Conformation-dependent ligand hot spots in the spliceosomal RNA helicase BRR2
Source: Acta Crystallogr D Struct Biol. 2023 Apr 28;79(Pt 4):304–17. doi: 10.1107/S2059798323001778 (PMC10071561; doi:10.1107/S2059798323001778)
Supplement: Supplementary file 1 [file d-79-00304-sup1.pdf]

STRUCTURAL BIOLOGY

Volume 79 (2023)

Supporting information for article:

Conformation-dependent ligand hot spots in the spliceosomal RNA helicase BRR2

Karen Vester, Alexander Metz, Simon Huber, Bernhard Loll and Markus C. Wahl

Table S1 Sources of the Fragments.

Fragment number	Source	NSC or catalog number
1	NCI	27259
2	NCI	28593
3	NCI	30143
4	NCI	78438
5	NCI	114955
6	NCI	125201
7	NCI	125210
8	NCI	135258
9	NCI	135784
10	NCI	141847
11	NCI	205947
12	NCI	209911
13	NCI	331994
14	NCI	406839
15	NCI	675198
16	NCI	675213
17	NCI	749154
18	NCI	4976
19	NCI	5863
20	NCI	7618
21	NCI	10929
22	NCI	15404
23	NCI	26354
24	NCI	45357
25	NCI	65057
26	NCI	119949
27	NCI	281325
28	NCI	401252
29	NCI	403677
30	In-house library	-
31	In-house library	-
32	In-house library	-
33	In-house library	-
34	In-house library	-
35	MolPort/Vitas-M	8006-5398
36	MolPort/ChemDiv	C607-0922
37	MolPort/ChemDiv	C607-0923
38	MolPort/Enamine	EN300-58198
39	MolPort/Enamine	Z1431002553
40	MolPort/Enamine	EN300-21442
41	MolPort/Enamine	Z166703618
42	MolPort/Specs	AN-329/42607819
43	MolPort/Vitas-M	STK864268
44	MolPort/Vitas-M	STL361683
45	MolPort/Vitas-M	STK782837
46	MolPort/Vitas-M	STK074818
47	MolPort/Vitas-M	STK085297
48	MolPort/Bionet	MS-10361
49	NCI	55259
50	NCI	300266
51	NCI	637723
52	NCI	376
53	NCI	125224
54	NCI	157600
55	NCI	201968
56	NCI	300395
57	NCI	376760

58	NCI	613272
59	NCI	131872
60	NCI	302043
61	NCI	348081
62	NCI	302082
63	NCI	55356
64	NCI	174536
65	NCI	642034
66	NCI	648616
67	NCI	637724
68	NCI	35600
69	NCI	637722
70	NCI	86089
71	NCI	211408
72	NCI	209954
73	NCI	209930
74	NCI	20638
75	NCI	20631
76	NCI	113534
77	NCI	528513
78	NCI	169595
79	NCI	139458
80	NCI	163639
81	NCI	372533
82	NCI	163636
83	NCI	163635
84	NCI	163638
85	NCI	163634
86	NCI	169590
87	NCI	268700
88	NCI	634166
89	NCI	373853
90	NCI	376759
91	NCI	373858
92	NCI	36945
PC	MolPort/Specs	AN-329/42613600

Table S2 Alignment of pockets of the hBRR2^{T1}-hJab1$^{\Delta C}$ complex and of isolated hBRR2^{T1}.

Binding site in the hBRR2^{T1}-hJab1$^{\Delta C}$ complex	Aligned domains of hBRR2^{T1} and the hBRR2^{T1}-hJab1$^{\Delta C}$ complex	RMSD [Å]
Pocket 1	WH (NC), HB (NC), RecA2 (CC)	4.12
Pocket 2	RecA1 (NC)	0.49
Pocket 3	RecA2 (CC), WH (CC)	0.66
Pocket 4	RecA1 (CC)	0.51
Pocket 5	RecA1 (NC), WH (NC)	0.73
Pocket 6	IG (NC) (omission of Jab)	0.44

The hBRR2^{T1} structure (PDB ID 4F91) was aligned to the hBRR2^{T1}-hJab1$^{\Delta C}$ complex structure (PDB ID 6S8Q) *via* the indicated domains involved in pocket formation of the ligands identified for the hBRR2^{T1}-hJab1$^{\Delta C}$ complex.

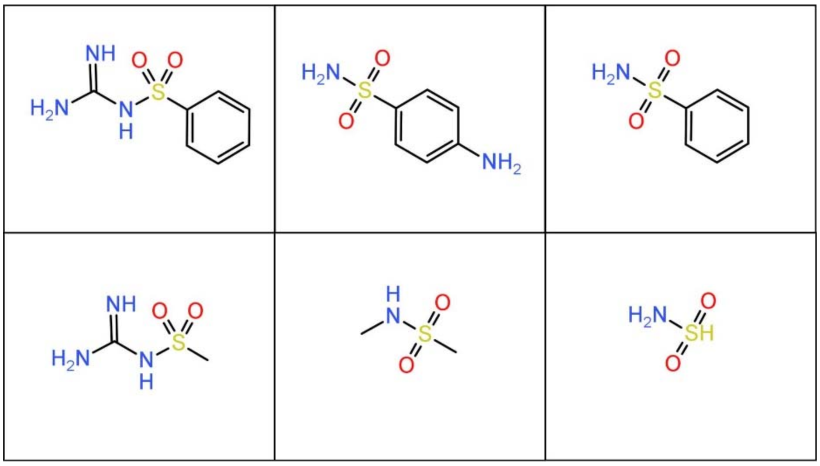

Figure S1 Six substructures derived from sulfaguanidine were used as templates for structure-guided docking. The substructures were each excised from the three-dimensional pose of sulfaguanidine in the hBRR2^{T1}-sulfaguanidine crystal structure (Fig. 1*b*) without modification of the respective three-dimensional coordinates. The structure images were prepared with DataWarrior (Sander *et al.*, 2015).

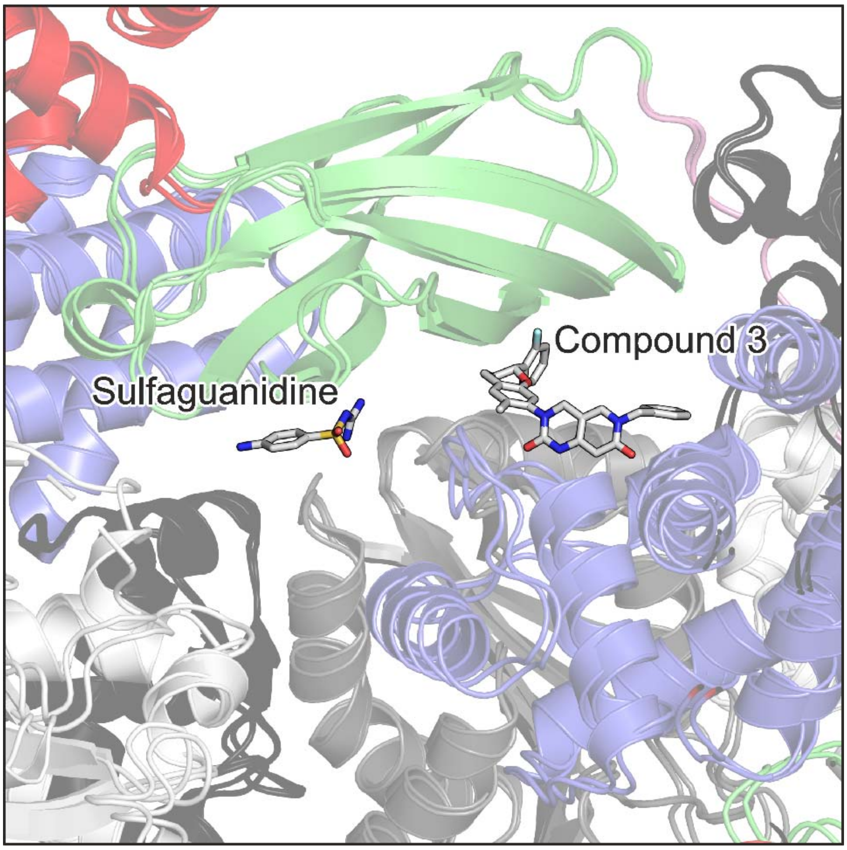

Figure S2 Comparison of the sulfaguanidine binding site with the binding site of a known hBRR2 inhibitor. The structure of hBRR2^{T1} in complex with sulfaguanidine was aligned to the structure of hBRR2^{T1} in complex with the previously described inhibitor, compound **3** of (Iwatani-Yoshihara *et al.*, 2017), which was subsequently elaborated to compound **33a** of (Ito *et al.*, 2017). The binding site of sulfaguanidine at the interface of the helicase cassettes is at a distance of around 9 Å to the position of compound **3** of (Iwatani-Yoshihara *et al.*, 2017).

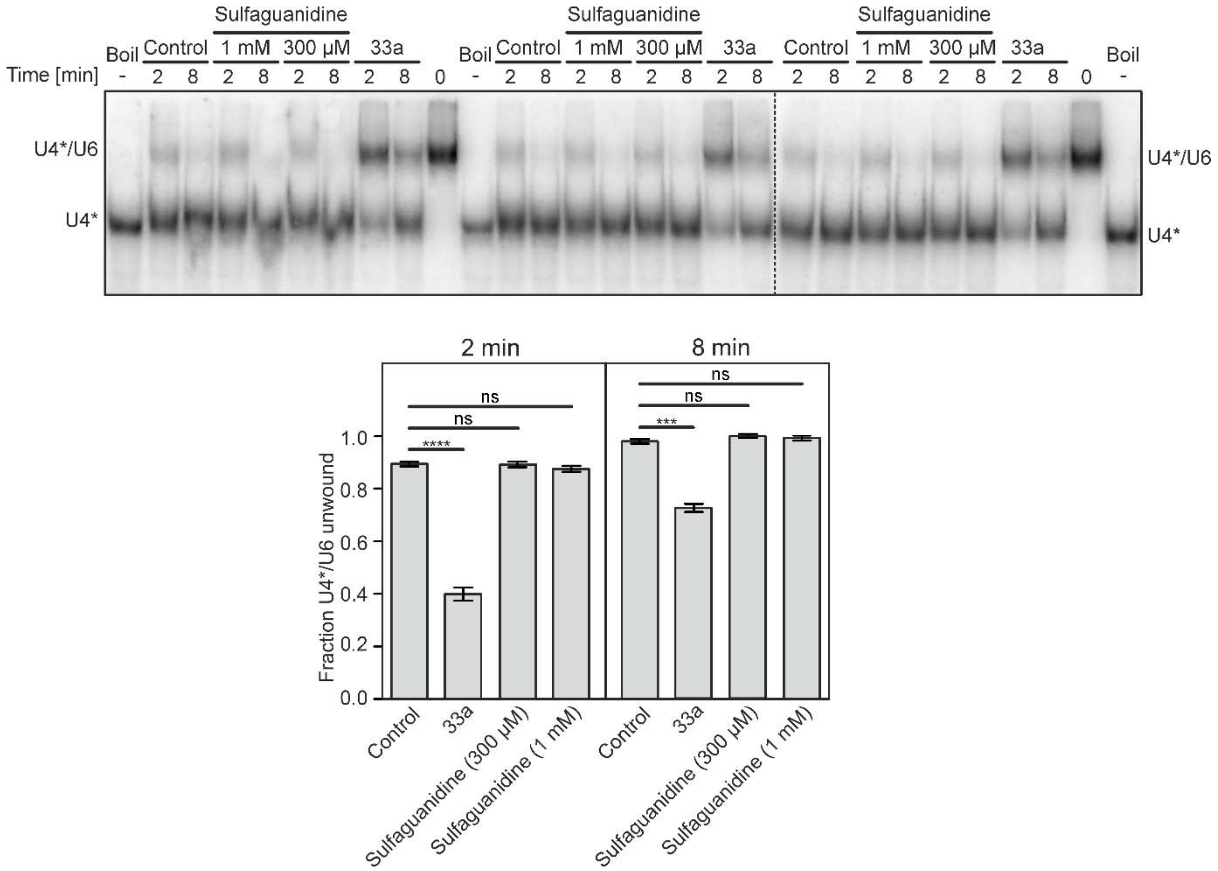

Figure S3 Upper panel, autoradiograms of non-denaturing gels monitoring single time point (2 min or 8 min) hBRR2[T1]-mediated U4*/U6 di-snRNA unwinding with DMSO (control) or with compound **33a** of (Ito *et al.*, 2017) or with sulfaguanidine (300 µM or 1 mM). The dashed line separates samples analysed on different gels. U4*, [^{32}P]-labelled U4 snRNA. Compound **33a** of (Ito *et al.*, 2017) was used as a positive control for a known hBRR2 inhibitor at a concentration of 1 mM. Boil, boiled U4*/U6 di-snRNA substrate, loaded as a control for completely separated strands. Time points 0, samples before the start of the reaction. Lower panel, quantification of the data in the upper panel. Data represent means +/- SD of n = 3 technical replicates. Significance indicators: ns not significant; ***, p ≤ 0.001; ****, p ≤ 0.0001.

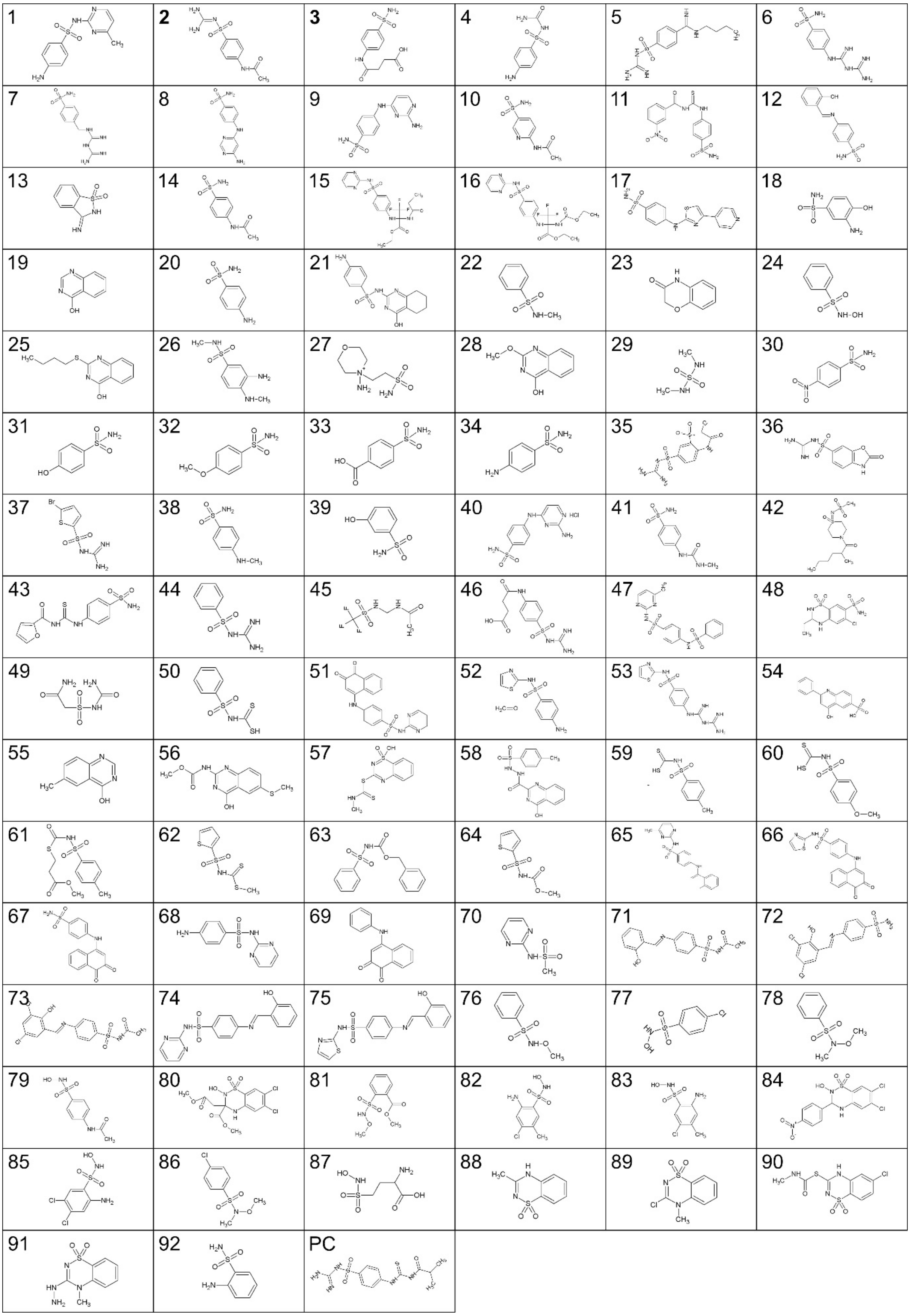
1
2
3
4
5
6
7
8
9
10
11
12
13
14
15
16
17
18
19
20
21
22
23
24
25
26
27
28
29
30
31
32
33
34
35
36
37
38
39
40
41
42
43
44
45
46
47
48
49
50
51
52
53
54
55
56
57
58
59
60
61
62
63
64
65
66
67
68
69
70
71
72
73
74
75
76
77
78
79
80
81
82
83
84
85
86
87
88
89
90
91
92
PC

Figure S4 Fragment structures. The structures of 93 sulfaguanidine analogues (obtained from NCI, MolPort and in-house libraries) are shown. PC, precipitated compound (largely insoluble in 100 % DMSO). Structures were generated with ACD/ChemSketch version 12.01.

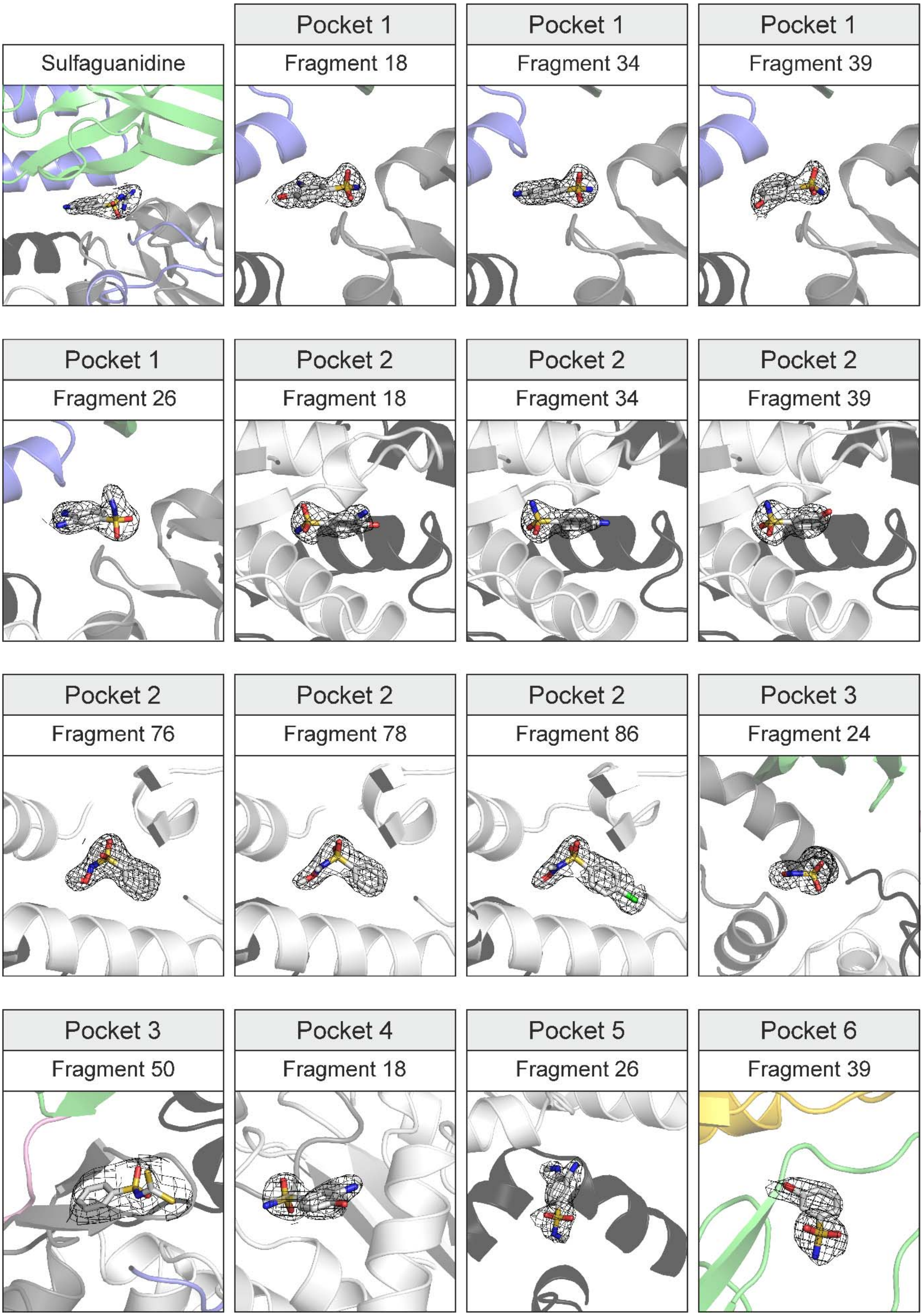
Sulfaguanidine
Pocket 1
Fragment 18
Pocket 1
Fragment 34
Pocket 1
Fragment 39
Pocket 1
Fragment 26
Pocket 2
Fragment 18
Pocket 2
Fragment 34
Pocket 2
Fragment 39
Pocket 2
Fragment 76
Pocket 2
Fragment 78
Pocket 2
Fragment 86
Pocket 3
Fragment 24
Pocket 3
Fragment 50
Pocket 4
Fragment 18
Pocket 5
Fragment 26
Pocket 6
Fragment 39

Figure S5 Electron densities of the fragment hits. $2mF_o$-DF_c electron densities covering the bound fragments are shown as meshes at a contour level of 1σ.

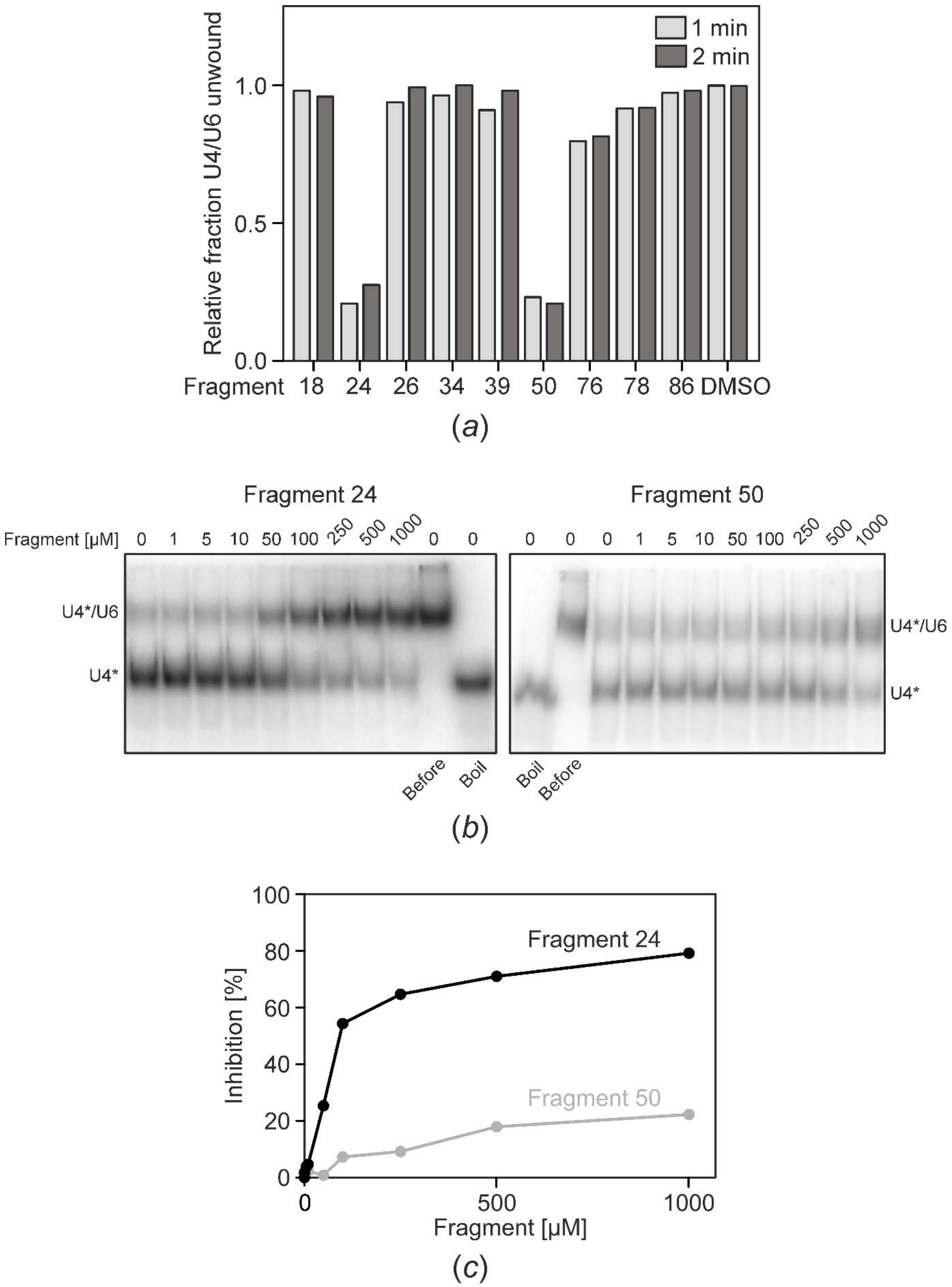

Figure S6 hBRR2^{T1}-mediated U4*/U6 di-snRNA unwinding in the presence of fragments. (*a*) Unwinding of the U4*/U6 di-snRNA substrate by hBrr2^{T1} was monitored at single time points (1 min

or 2 min) in the presence of 1 mM of fragments **18**, **24**, **26**, **34**, **39**, **50**, **76**, **78** or **86**. U4*, [^{32}P]-labelled U4 snRNA. The ratio of unwound U4*/U6 di-snRNA was calculated from quantified radioactive band intensities and normalized to the respective values observed in the presence of DMSO. (*b*) Autoradiograms of non-denaturing gels monitoring hBRR2^{T1}-mediated U4*/U6 di-snRNA unwinding (2 min time points) at increasing concentrations of fragments **24** (left) or **50** (right). Final concentrations of fragments (0-1000 μM) are indicated above the gels. Before, control samples without starting the unwinding reaction. Boil, boiled U4*/U6 di-snRNA substrate, loaded as a control for completely separated strands. (*c*) Quantification of the data shown in (*b*) relative to the DMSO control for evaluation of IC_{50} values.
